# Unsuccessful Attempt to Remove a Fibrous Tumor of the Anterior Wall of the Uterus—Death from Peritonitis

**Published:** 1873-03

**Authors:** A. Reeves Jackson

**Affiliations:** Surgeon-in-Chief of the Woman’s Hospital, of the State of Illinois; 785 Michigan Ave.


ARTICLE III.—*Unsuccessful Attempt to Remove a Fibrous Tumor of the Anterior Wall of the Uterus—Death from Peritonitis.* By A. REEVES JACKSON, M.D., Surgeon-in-Chief of the Woman's Hospital, of the State of Illinois.

I was summoned to visit Abigail R. Hall, at Janesville, Wisconsin, August 26th, 1870, and obtained the following history :

The patient is forty-seven years of age, unmarried, and has a strongly-marked strumous appearance, with very light hair and complexion. She commenced menstruating at the age of thirteen, the discharge being regular and painless down to the age of seventeen. At that time, in attempting to lift a heavy weight, she felt a sudden pain in the lower part of the abdomen. Subsequently menstruation became habitually profuse, but recurred at regular intervals and was still painless. At the age of twenty-four, in alighting from a carriage, she sprang heavily from the wheel to the ground, and again felt a severe pain in the hypogastric region. A physician who examined her at that time stated that there was prolapsus and enlargement of the womb. Since that occurrence the patient has observed a slowly increasing fullness in the lower abdomen. Menstruation continued rather profuse, and leucorrhœa was present throughout the intermenstrual period. At the age of forty-one she had an attack of pelvic cellulitis apparently, which was followed by a copious discharge of pus through the rectum. Since that event the hypogastric enlargement has been more distinct, and has continued to increase, although not steadily. There have been times when it seemed to become less, and again at others would be attended with so much fullness as to occasion oppression of the breathing. These periods of unusual enlargement were associated with œdema of the lower extremities. The size of the abdomen was always lessened during and immediately after the menstrual flow. From August last to the following November there was no sanguineous discharge whatever, and during that period her general health was notably better than it had been for years previously. From November to March menstruation appeared irregularly at short intervals, and in diminished amount. It then ceased again entirely until three weeks ago—a period of nearly five months—during which time she grew rapidly larger, and the lower extremities became much swollen, the left one especially. Three weeks ago a sanguineous discharge commenced and continued two weeks, during which time the dropsical symptoms subsided.

The present appearance of the patient is that of a woman who has undergone much suffering. She is pallid, has a rather stooping gait, and a worn expression of countenance.

An examination of the abdomen reveals the presence of a very evident tumor reaching from the navel to the pubic ridge, and traceable into the pelvis. It is firm, smooth, rounded, symmetrical, and non-fluctuating, resembling in shape and position the gravid womb, except in being much harder. In the right iliac region there is a spot that is tender under pressure; with this exception the tumor is painless.

The vagina is small, but presents no other noticeable characteristic. The cervix uteri occupies its normal position, but is shortened, and, the anterior lip especially, much thickened. The os is healthy in every respect. Under conjoined manipulation, with one finger of the left hand pressed against the cervix, and the other hand making simultaneous pressure upon the abdominal tumor downwards into the pelvis and rolling it from side to side, the cervix appears to move consentaneously with the tumor. A probe can be introduced only to the depth of about three-quarters of an inch.

Introduce a sponge tent as far as possible through the os uteri. This is done at eleven o'clock in the evening.

August 27th. At ten o'clock A. M. remove the tent and make another attempt to introduce a sound, with the result of finally passing it in about an inch and a half. In entering, it takes a direction backwards towards the rectum. When in this position motion is distinctly transmitted to its handle through the abdominal wall. I now pass an elastic bougie mounted upon a stout wire into the os uteri, and holding the distant extremity of the wire firmly with one hand, push the bougie onward into the cavity of the cervix with the other. It passes readily in to the depth of seven inches, thus showing the depth of the cavity but not its direction. From the course taken by the sound I suppose this to be backwards, but in order to remove any doubt on this point I introduce a finger into the rectum and distinctly detect the presence of the bougie through the recto-vesical septum. The diagnosis is a fibrous tumor of the anterior wall of the uterus.

I advise the external use of iodine over the site of the tumor, and internally, muriate of ammonia in ten grain doses three times a day, the treatment to be continued many months; and encourage the patient by telling her that as the climacteric is now at hand, the tumor may diminish in size, or at least prove comparatively innocuous.

November 11th. To-day I visit Miss Hall at the Woman's Home, she having come to the city for treatment.

Her condition has not changed materially since I last saw her. She seems scarcely so well nourished, however, and states that the tumor is more painful, and that hemorrhages have been more frequent and at times very profuse. There is a considerable increase in the size of the abdomen, and the tumor has grown; the latter now extends to a point an inch and a half above the umbilicus, although a vaginal examination reveals no change from her former condition. She has not been able to take the sal ammoniac owing to the fact that it produced nausea. Sometimes she had taken it for three or four days consecutively, but so much impairment of appetite and irritability of stomach followed its use that she was obliged to discontinue it. Iodine in the form of Ung. Iodin. Comp. had been used regularly.

Inasmuch as there is no hemorrhage present, and no other symptom of an urgent character, I conclude simply to watch the case a few weeks before determining the question of surgical interference.

December 12th. The patient's general health is clearly giving way, and it becomes evident that she cannot live under existing circumstances more than a few months. There appears to be no other disease present than the uterine tumor; and the removal of this offers the only hope of warding off the impending result. The patient and her friends are fully apprized of the dangerous nature of the operative procedures necessary for the removal of the growth, and of the unpromising character of the case, and after due consideration decide to have the attempt made. The patient herself is especially anxious, and very sanguine of a successful result.

January 3rd, 1871. During the past three weeks the patient has been taking tonic medicines and using other various means for the purpose of getting her general health in as good condition as possible prior to commencing operations. Notwithstanding this, however, she has lost strength rather than gained it. The tumor has been the seat of so much pain that she has been able to get very little sleep. Thinking it unsafe to delay any longer I introduce a sponge tent into the os uteri as far as possible.

January 6th. Have introduced a sponge tent every twenty-four hours, the size being each time larger than that of the one which preceded it. Remove the third one to-day, and am enabled to introduce the finger as far as the second joint. The tumor is now felt starting abruptly from the anterior wall of the cervix at a point about one inch above the os uteri.

Introducing a speculum, I divide the cervix, right and left, to within a quarter of an inch of the vaginal junction, by means of scissors. This is followed by pretty copious bleeding, which promptly ceases on the withdrawal of the speculum. Order ten grains of powd. ergot to be given every four hours.

January 9th. The ergot has caused much nausea, but produced no effect upon the uterus. I introduce a long-handled probe-pointed bistoury, guided by the finger, as high as possible through the now widely open os uteri, and make a tolerably deep incision into the capsule of the tumor. A small amount of hemorrhage follows the operation, and it ceases as before on the closure of the vagina. Order wine of ergot instead of the powder.

January 12th. The vin. ergotæ has acted more pleasantly and more efficiently, producing less nausea and exciting pretty severe contractile pains. The tumor and womb have settled lower down in the pelvis. After introducing a finger as a guide into the cervix, I pass an instrument with a concealed knife—resembling an urethrotome—to the highest attainable point, and projecting the blade into the capsule of the tumor, withdraw the instrument, pressing, as I do so, the blade firmly into the growth. In this way I make an incision about four inches long and nearly a half inch in depth. A very trifling amount of blood follows. The ergot to be continued every four hours.

January 26th. On the 21st there appeared a sanious gray and somewhat offensive discharge which contained small threads of putrid substance. The patient's appetite continues fair, but she seems weaker and does not sleep well. Her pulse which lately has been from 90 to 100, has risen to 120, and she is peevish and fretful—a condition of temper more noticeable from the fact that naturally her disposition is extremely amiable.

I make another incision into the capsule of the tumor, and order a suspension of the ergot, giving, instead, a quarter grain of sulph. morph. with two grains sulph. quinia every six hours; also a glass of sherry wine with each meal.

January 30th. To-day the patient seems better. The grayish discharge is largely increased in quantity and highly offensive. Masses of the now softened and disintegrated tumor have been coming away, and the growth felt through the abdominal wall has become softened in places and imparts a feeling of semi-fluctuation. There is more marked tenderness in the right iliac region, which is greatly increased under pressure. She is less irritable, has slept better, and there is some improvement of the appetite.

February 1st. Was unable to visit my patient yesterday. To-day find her with well marked symptoms of peritonitis. The pulse is small, rapid and sharp; tongue dry and brown; abdomen tympanitic and very tender—the greatest tenderness still being on the right side, respiration short and shallow. Her countenance has a worn, anxious expression, and there is almost constant jerking of the forearms and wrists. She lapses frequently into a condition of semi-consciousness, during which she mutters incoherently.

Order hot turpentine stupes to be applied to the belly, and a quarter grain of sulph. morph. every three hours. Leave her at 11 A. M.

In the evening she is attacked with vomiting and great restlessness, with coldness of the extremities. She dies at half-past nine o'clock in the evening of February 2nd.

The post-mortem examination revealed extensive inflammation of the peritoneum, particularly in the neighborhood of the right side of the womb, involving the right ovary, which was thoroughly disintegrated and covered with lymph and pus. The tumor was found to be imbedded in the anterior wall of the womb, and its substance throughout showed signs of decomposition, especially in the neighborhood of the incisions. The extreme lower portion of the tumor was soft and shreddy and thoroughly phagedenic.

The incisions which had been made before death were now continued to the full length of the uterine canal, and then with the fingers alone the capsule was peeled off from the tumor almost as readily as one would remove the rind from an orange. The weight of the tumor after removal from its capsule was four and a quarter pounds.

*Remarks.* Our successful cases are not by any means always the most valuable and instructive, and the case just related has been especially useful to the writer in teaching the necessity of caution in the treatment of uterine fibroids.

The time is not very long past when intramural tumors of the uterus were regarded as wholly unamenable to surgical treatment. And even now operative measures are restricted by many excellent practitioners to means designed to control the hemorrhages which are so frequent an accompaniment of these growths, and which form the chief element of danger. Others, however, have considered it justifiable to employ medical means for their removal in cases where their presence places the life of the patient in jeopardy.

These means for radical cure are now recognized as five in number, namely: 1. Incising the cervix uteri; 2. Incising the capsule of the tumor; 3. Mutilating the tumor by various means—usually termed "gouging"; 4. Enucleation; 5. Avulsion.

The first two named were both used in the case of Miss Hall, and commonly I regard it as best to combine them. The incision of the os uteri is based upon a theory which originated with Mr. Baker Brown that "the division of the os and cervix uteri permits the fibres of the body of the uterus to contract upon the contained tumor, and thereby to compress the vessels and check the hemorrhage." The operation, originally used only for the purpose of checking the bleeding, was found in some cases to be followed by expulsion of the tumor through the enlarged os, and thence came to be used as a means of inducing such a result. Dr. McClintoch, of Dublin, has practiced the operation very extensively, and as he alleges, with a good degree of success.

Incising the tumor has been a favorite operation with Dr. W. L. Atlee, of Philadelphia, for many years, and as early as 1853 he published for private distribution a valuable essay on "The Surgical Treatment of certain Fibrous Tumors of the Uterus," in which a number of cases were detailed in which the plan in question proved successful. In that essay, Dr. Atlee called especial attention to a fact of great importance in the treatment of patients afflicted with the class of tumors under consideration. It was "*that the excessive hemorrhages which sometimes occur, arise not from the uterus itself, but from the vessels of the membrane which cover the tumor.* And the practice which he based upon this fact was "*During hemorrhage to pass the bistoury along the vagina into the cavity of the uterus, and make a very free incision into the most exposed part of the tumor.*" This appears at first sight to be a startling mode of practice, but from considerable experience I can bear testimony to its efficacy and safety. The incision is followed by a small gush of blood, and the hemorrhage which may have persisted for many days, is almost instantaneously arrested.

The success of this procedure as a radical means of cure depends, doubtless, upon the fact that these tumors are endowed with very feeble vitality, and that it needs but little disturbance to interrupt their nutrition. The proper uterine tissue does not enter into their composition; it is merely pushed aside by the neoplasm, and forms around the latter a covering or capsule. And so loose is the connection between the capsule and the tumor, that when a free incision is made in the former, the contractile power of the uterus is sufficient in many instances to push the tumor through the opening and finally expel it.

There were some circumstances in the case of Miss Hall which caused me to hesitate before resorting to surgical means for the removal of the tumor. They were, the large size of the tumor, the fact of its being the seat of pain, and the failure of the general health. These symptoms were all unfavorable to success. So long as fibrous tumors remain small, hard, painless, insensible to pressure, and do not interfere with any important function of the neighboring organs, or give rise to hemorrhage, they do not greatly impair the general health. And this is the time when they should be removed, provided they threaten to produce these results; for at this time their removal may result in complete cure. But after they begin to soften, are subject to attacks of acute pain, and are tender to the touch—symptoms denoting the degeneration of the growth; and when, in addition to these symptoms, there are also anasarca, ascites, emaciation and pallor, indicating the failure of the general system, it is usually too late for a successful operation.

Now some of these very unfavorable symptoms were present in this case. But it was evident that without interference death was imminent, and there was at least a possibility that an operation might result in cure. I think, too, that I was influenced, perhaps more than I should have permitted myself to be, by the entreaties and hopefulness of the patient. So soon as she learned that an operation offered her any prospect of relief, however small, she begged to have it done. I do not wish to be understood as offering this circumstance as a justification of a course of conduct my conscience did not approve, or my judgment did not sanction; on the contrary, I regarded the operation as justifiable under all the circumstances, and had I refused to interfere and the patient had died unrelieved, as she unquestionably would have done very soon, I should have felt that I had been recreant to my duty.

785 Michigan Ave.

